# Safety of transcutaneous electrical stimulation potentiating recovery in acute spinal cord injury syndromes: a trial protocol

**DOI:** 10.3389/fnins.2026.1875365

**Published:** 2026-07-22

**Authors:** Anastasia Keller, Prakruthi Amar Kumar, Xuan Bradfield, Debra Hemmerle, J. Russell Huie, H. E. Hinson, Rajiv Saigal

**Affiliations:** 1 Brain and Spinal Injury Research Center, Department of Neurological Surgery, University of California, San Francisco, San Francisco, CA, United States; 2 Department of Neurology, University of California, San Francisco, San Francisco, CA, United States

**Keywords:** acute spinal cord injury, clinical trial, intensive care unit, safety, spinal cord stimulation

## Abstract

**Background and objectives:**

Spinal cord injury (SCI) often results in permanent paralysis, leading to substantial long-term disability and loss of quality-adjusted life years. Strategies to preserve neural function in the hyperacute phase are needed. Transcutaneous spinal cord stimulation (tSCS) improves neurological function in chronic SCI. Earlier implementation in acute SCI may preserve or restore function and improve recovery.

**Methods:**

STEP-RAISE is a Phase 1/2 prospective clinical trial evaluating the safety and preliminary efficacy of tSCS in acute moderate-to-severe traumatic SCI. Electrical current will be delivered to the lumbosacral spinal cord using the investigational ARC-Ex device (ONWARD Medical) via surface electrodes over the thoracolumbar region. Phase 2 is a randomized, triple-blinded, placebo-controlled trial designed to assess treatment effects. Safety outcomes include hemodynamics, spinal cord perfusion pressure, and skin reactions. Efficacy will be assessed using the International Standards for Neurological Classification of Spinal Cord Injury (ISNCSCI) examination with electromyography at baseline, during stimulation, and at 6-month follow-up. Biomarkers include neuronal (UCH-L1) and glial (GFAP) markers and exploratory proteomics in blood and cerebrospinal fluid collected prior to stimulation and at discharge.

**Discussion:**

The STEP-RAISE trial evaluates safety and preliminary efficacy of non-invasive tSCS initiated within 72 h of SCI to improve sensorimotor recovery. Neurophysiological and patient-reported outcomes will characterize recovery and assess whether early tSCS augments functional recovery. This study is designed to estimate treatment effects and inform subsequent trials in acute SCI. Planned enrollment includes 5 participants in Phase 1 and 10 in Phase 2.

**Clinical trial registration:**

ClinicalTrial.gov: NCT07090473; first posted on 07/29/2025, first open to enrollment 04/2026. https://clinicaltrials.gov/study/NCT07090473

## Introduction

### Background and rationale

Spinal cord injury (SCI) is a severe neurologic condition for which no established neuroprotective therapy exists in the acute phase. In the United States, more than 100,000 individuals sustain SCI each year, and over 2 million people live with chronic disability related to these injuries (Safdarian et al., 2023). Acute SCI (aSCI) commonly leads to persistent motor, sensory, and autonomic impairments, resulting in substantial long-term morbidity and societal burden. Current acute management is largely supportive and includes surgical decompression and maintenance of adequate spinal cord perfusion (Shank et al., 2019; Karsy and Hawryluk, 2019). Pharmacologic strategies such as high-dose corticosteroids remain controversial and are used inconsistently (Pointillart et al., 2000; Matsumoto et al., 2001). Despite early rehabilitation, recovery of voluntary movement below the level of injury is often limited. Patients are also vulnerable to systemic complications related to immobility and autonomic dysfunction, including cardiovascular instability (Wecht et al., 2020), immune dysregulation (Schwab et al., 2014), and respiratory impairment (Rosales-Antequera et al., 2022). The initial hospitalization, lasting 11 days on average, represents a unique window during which secondary injury processes and physiologic deconditioning may further worsen outcomes (Team, S. C., 2023).

There is, therefore, a significant unmet need for interventions that can be initiated early after injury to preserve neural function and mitigate downstream complications. Electrical stimulation has been investigated as a potential neurotherapeutic strategy in SCI. In preclinical models, externally applied electric fields influence axonal orientation and cellular behavior (Marsh and Beams, 1946; Patel and Poo, 1982). Studies using oscillating field stimulation applied directly to injured spinal cord tissue have demonstrated improved locomotor recovery in animals, associated with greater tissue preservation, enhanced oligodendrocyte survival and differentiation, axonal remyelination, and reduced glial scar formation (Bacova et al., 2022; Zhang et al., 2015). These findings suggest that electrical stimulation can modulate biological processes relevant to secondary injury.

Activity-patterned stimulation paradigms further indicate that electrical input may influence network-level physiology after trauma (Taccola et al., 2020). In rodent models of acute contusion SCI, stimulation designed to approximate physiological sensorimotor activity has been associated with restoration of motor-evoked potentials compared with sham stimulation (Taccola et al., 2020). Although mechanisms remain incompletely defined, proposed effects include stabilization of network excitability and activity-dependent neurotrophic signaling (Taccola et al., 2020). Collectively, preclinical data support the premise that electrical stimulation can alter both cellular and circuit-level responses after SCI.

Only limited pilot studies in humans after acute SCI with implanted stimulators demonstrate safety and signs of potential efficacy (Shapiro et al., 2005). Clinical translation of spinal cord stimulation has advanced most in chronic SCI. Epidural lumbosacral stimulation has enabled recovery of sensorimotor and some autonomic functions in selected individuals with longstanding paralysis, demonstrating that spinal networks below the lesion retain capacity for functional modulation (Aslan et al., 2018; Harkema et al., 2011; Angeli et al., 2014; Calvert et al., 2026; Phillips et al., 2025; Shackleton et al., 2023; Singh et al., 2023). Non-invasive transcutaneous spinal cord stimulation (tSCS), delivered through surface electrodes, avoids surgical implantation, and has been associated with improvements in motor performance, trunk control, and standing or stepping ability when combined with rehabilitation in chronic SCI (Inanici et al., 2021; Gerasimenko et al., 2015; Sayenko et al., 2019; Rath et al., 2018). A proposed mechanism is increased excitability of lumbosacral spinal networks, enhancing responsiveness to residual descending and sensory inputs (Gerasimenko et al., 2015).

tSCS has demonstrated a favorable safety and tolerability profile in rehabilitation settings, including pediatric populations (Keller et al., 2021; Amirova et al., 2025; Lucas et al., 2025; Singh et al., 2024). Its non-invasive nature, feasibility, and physiological effects make it a candidate intervention for earlier use after injury. Based on preclinical evidence and clinical data from chronic SCI, we designed a clinical trial to evaluate whether non-invasive lumbosacral tSCS applied distal to the lesion site may be safely initiated during acute hospitalization after SCI and show signals of biological and functional impact, with the goal of informing strategies to improve neurologic recovery trajectories.

### Objectives

This study has three main objectives. First, test the safety of tSCS applied below the level of injury to the lumbosacral spinal cord after acute spinal cord injury, starting 72 h after injury. We hypothesize that there will be no adverse events associated with acute application of tSCS. Second, demonstrate the proof-of-principle that tSCS application in the acute phase post-injury can potentiate recovery. We hypothesize that tSCS can improve volitional movement and/or sensation acutely after SCI as measured by an improved lower extremity motor score in the presence of tSCS. Third, identify treatment-related mechanistic biomarkers (exploratory). We hypothesize that there will be fluid-based biomarkers that will change in the setting of tSCS receipt.

## Methods

### Patient and public involvement

In accordance with Congressionally Directed Medical Research Programs (CDMRP) requirements, two individuals with lived experience with SCI were invited to serve as community representatives for this clinical trial. Their feedback was solicited to inform study execution. The study design, protocol, and informed consent materials were reviewed and discussed during a dedicated meeting and shared with the community representatives to incorporate their input.

### Trial design

**Figure 1:**
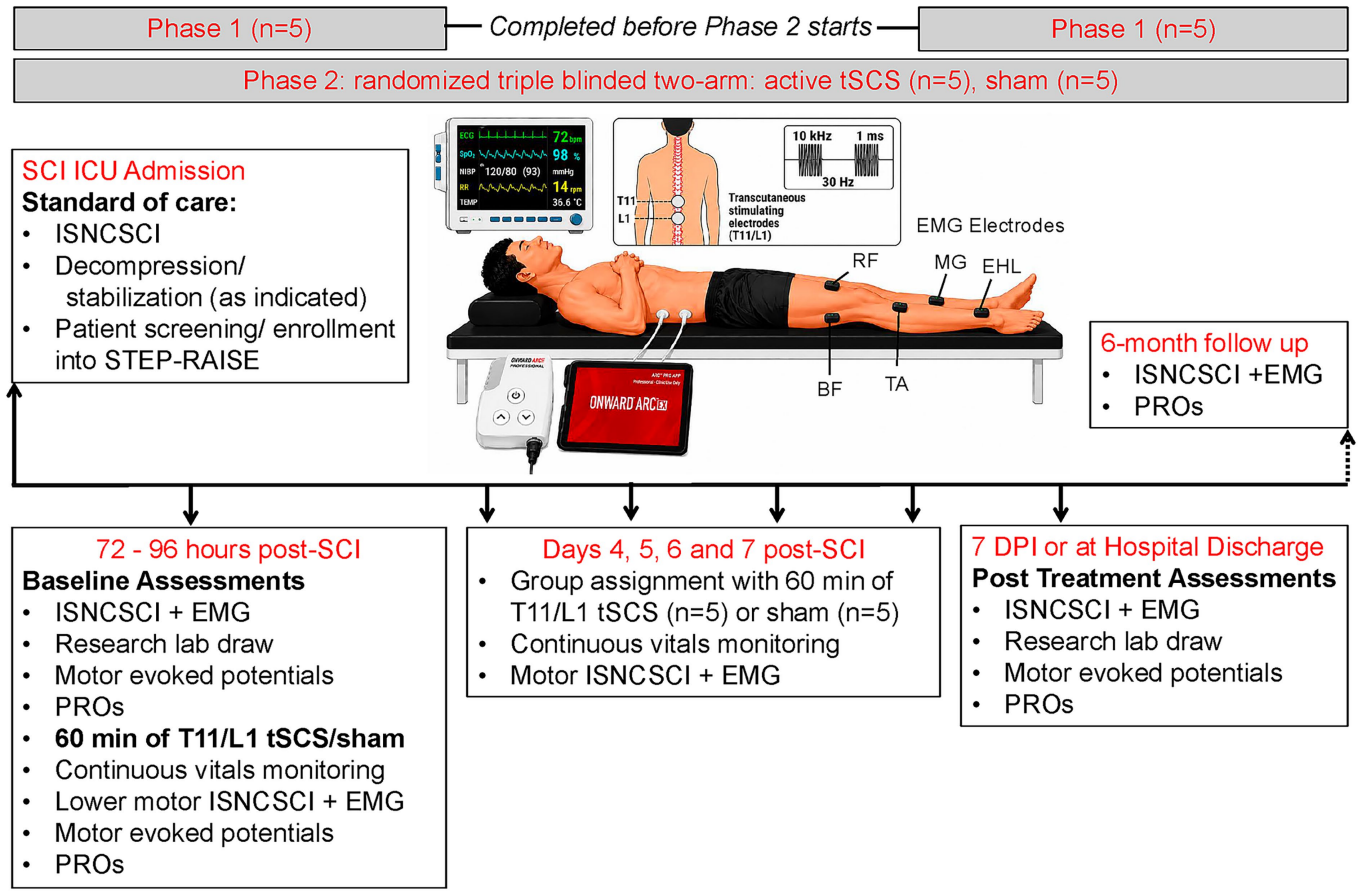
Study design and transcutaneous spinal cord stimulation paradigm in acute spinal cord injury. Schematic representation of the two-phase clinical trial conducted in the acute spinal cord injury (SCI) setting. Phase 1 (*n* = 5) consists of an open-label safety assessment during a single 60-min tSCS application, followed by Phase 2 (*n* = 10), a randomized, participant- and assessor-blinded comparison of active versus sham transcutaneous spinal cord stimulation (tSCS). Participants are enrolled upon admission to the intensive care unit and undergo baseline assessments starting at 72 h post SCI within the first 96 h, including International Standards for Neurological Classification of Spinal Cord Injury (ISNCSCI) examination, lower extremity electromyography (EMG), motor evoked potentials, blood and cerebrospinal fluid draws for biomarker assessment, and patient-reported outcomes (PROs). In Phase 2, participants receive daily tSCS or sham stimulation at T11/L1 for 60 min for a total of 5 sessions. Continuous vital sign monitoring is performed during stimulation sessions. Follow-up assessments, including neurological, neurophysiological, and patient-reported measures, are conducted at 7 days post-injury and at 6 months for both Phase 1 and Phase 2 participants. RF—rectus femoris, BF—biceps femoris, MG—medial gastrocnemius, TA—tibialis anterior, EHL—extensor hallucis longus, DPI - days post injury.

We will conduct a Phase 1 and Phase 2 clinical trial (Figure 1). Phase 1 is an open label study focused on determining safety of the initial 60 min of tSCS between 72 and 96 h after spinal cord injury (*n* = 5). Phase 2 is a randomized, placebo-controlled triple-blinded clinical trial with a parallel group assignment (active tSCS, *n* = 5 and sham tSCS *n* = 5) focused on determining safety and efficacy of tSCS for treatment of acute SCI. A total of 15 subjects will be enrolled across Phase 1 and Phase 2. A sham-controlled design was selected to isolate the physiological effects of electrical stimulation from nonspecific influences related to device application, participant expectations, investigator interaction, and increased clinical monitoring.

### Trial setting

This study will be conducted in the neurotrauma intensive care unit (ICU) at Zuckerberg San Francisco General Hospital and Trauma Center (ZSFG). All patients will be admitted to the ICU, and treated per standard of care according to hospital protocol for acute SCI.

### Eligibility criteria

**Figure 2:**
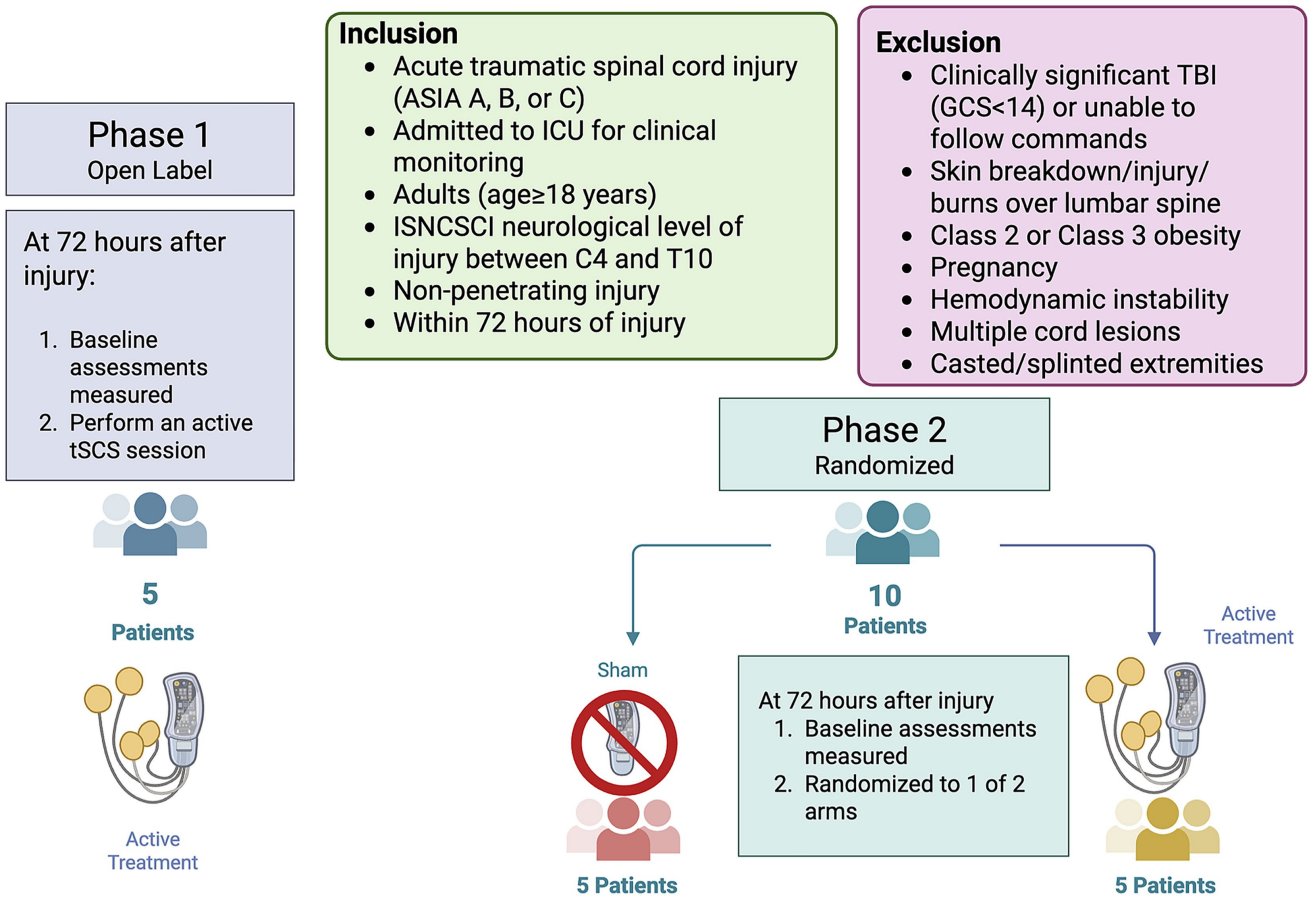
Eligibility criteria. Overview of the two-phase trial inclusion and exclusion criteria and participant allocation across open-label and randomized phases.

Patients will be screened for eligibility during the first 3 days of ICU stay based on the following eligibility criteria (Figure 2):

Inclusion criteria:

A subject will be eligible for enrollment in the study if the following criteria apply:

1 Written informed consent is obtained.
2 Acute traumatic cervicothoracic SCI that meets all of the following criteria:

- Acute SCI presenting to the hospital within 12 h of injury
- Traumatic non-penetrating SCI
- AIS Grade A, B, or C
- ISNCSCI neurological level of injury between C4 and T10

3 Aged ≥18 years
4 Admission to ICU

Exclusion criteria:

1 Subjects classified as AIS D or E
2 Penetrating SCIs or complete transection of the spinal cord
3 Pregnancy
4 Incarceration or police custody
5 Class 2 or Class 3 obesity
6 Any concomitant injury that, in the judgment of the Investigator, interferes with the procedures and examinations required by the study protocol, including but not limited to:

- Multiple spinal cord lesions
- Fractures requiring lower extremity casts or splints
- Skin breakdown or burns over the lumbar spine
- Profound hemodynamic instability
- Traumatic brain injury (defined by GCS < 14 at enrollment or inability to participate in exam)

### Intervention and comparator

Transcutaneous spinal stimulation will be delivered using ARC^EX^ device (ONWARD Medical), via skin surface electrodes placed over the spine targeting lumbosacral spinal cord at T10 and L1 using round 3.2 cm in diameter stimulating electrodes, and bilaterally on the iliac crests using rectangular, 7.5 × 13 cm reference electrodes during the intervention. Based on previous studies on safety and efficacy of tSCS, we will use biphasic waveforms with a burst frequency of 30 Hz, a carrier frequency of 10 kHz, with a pulse width of 1,000 μs (Gerasimenko et al., 2015; Keller et al., 2021; Moritz et al., 2024). Stimulation will be delivered continuously for 60 min once (for Phase 1 participants) and once a day for 5 consecutive days (for Phase 2 participants). The intensity of the stimulation will be increased gradually to the motor threshold intensity (Moritz et al., 2024) and lowered until motor responses subside if there are any. Increase in trunk/lower extremity muscle tone will be additionally monitored to assess whether the amplitude of stimulation required to reach motor threshold changes with days post injury + tSCS treatment for Phase 2 participants. If stimulation does not elicit any obvious motor responses due to spinal shock, the stimulation intensity will be chosen based on previously reported ranges of effective stimulation, including those observed in our ongoing clinical trial in patients with chronic low back pain (NCT05265000) which provides ranges of maximum tolerable stimulation intensities in individuals without SCI, who have full sensation. The stimulation parameters used in that study are identical to those proposed in STEP-RAISE, including waveform, pulse width, carrier frequency, and electrode configuration, allowing for adaptation of stimulation intensities if necessary. The specific stimulation parameters and optimal stimulation intensity will be left to the discretion of the investigators during this experimental treatment. A range of the investigated stimulation parameters will be systematically recorded and will be reported as part of the methodology for each participant. The stimulation will be delivered while the participant is lying supine in the ICU bed. The lower motor ISNCSCI exam will be performed within the last 30 min of stimulation to assess whether acute tSCS potentiates volitional lower extremity movement. Although physical therapy (PT) is provided to acute SCI patients, in this trial the goal is to assess whether tSCS alone can be delivered safely, and will not be combined with the standard of care ICU PT. Participants will be continuously monitored and asked to provide any verbal feedback regarding stimulation to ensure there is no pain or discomfort during treatment.

For sham stimulation (comparator), electrodes will be placed in the same location. The participant will be continually monitored and given similar verbal cues as with the stimulation trials, but no electrical current will be delivered. Participants in both arms will be informed that they may or may not feel the stimulation.

### Criteria for stopping the intervention

The intervention will be paused for significant hemodynamic changes, defined as a sustained (>15 min) increase or decrease in mean arterial pressure (MAP) of more than 30% from baseline or the last recorded stable value and is clinically symptomatic. It will be stopped if spinal cord perfusion pressure (SCPP) decreases to <65 mmHg for more than 15 min despite standard clinical management, including appropriate vasopressor titration. The intervention will also be paused for severe hemodynamic instability, such as persistent bradycardia, hypotension, or hypertension despite appropriate medical management, or for signs of autonomic dysreflexia, including sudden severe headache, flushing and profuse sweating above the level of injury, hypertension, bradycardia, anxiety, blurred vision, or nausea. Any serious adverse event deemed related to the intervention by the Principal Investigator or the safety monitor, or any unanticipated adverse device effect, will result in immediate suspension of the intervention pending further evaluation. If the patient experiences clinical deterioration that precludes participation (e.g., severe sepsis or coma), the intervention will be paused and reassessed. If the intervention is permanently discontinued, observational data collection will continue without further stimulation or sham trials until hospital discharge.

### Concomitant and post-trial care

Participation in the study will not alter provision of standard of care during or post-trial. Individuals who consent to participate in the study, will however, have additional experimental assessment and treatment procedures incorporated while they are in ICU. The additional assessments will include blood draws and testing of motor evoked potentials using tSCS. The additional treatment will include a single 60 min of tSCS for Phase 1 participants and 5 days of once daily 60 min of active tSCS or sham tSCS. The frequency of certain standard of care procedures, such as ISNCSCI to assess the neurological status of the participant will be increased to daily assessments. The adherence to intervention protocol will be directly monitored by the research staff who will conduct all experiments during the in-patient hospital stay. Any adverse events related to study procedures will be managed promptly by the clinical team according to institutional standards. Compensation for research-related injury will be provided in accordance with University of California, San Francisco institutional policies. No additional post-trial interventions are planned.

### Outcomes

Table 1 lists all trial outcome measures and the corresponding abbreviated statistical analysis plan.

**Table 1:** A list of outcome measures and the corresponding analysis plan.

Outcome measure	Description	Statistical analysis
Primary outcomes (safety)—Phase 1 and 2		
1. Incidence of adverse events (# of observed events/sessions)	Hemodynamic instability Skin irritation Patient condition deterioration Unanticipated adverse events	Data type: Count Data Statistical Test: Fisher’s Exact Test
Primary outcomes (efficacy)—Phase 2		
1. ISNCSCI lower motor scores	Immediate change in scores with acute tSCS Change in scores from baseline to post tSCS	Data Type: Integer Statistical Test: Repeated Measures Analysis of Variance
Secondary outcomes (efficacy)—Phase 1 and 2		
1 ISNCSCI sensory + motor scores	Change in scores from baseline to 7DPI or discharge	Data Type: Integer Statistical Test: Repeated Measures Analysis of Variance
2. Spinally motor evoked potentials (MEPs)	MEP present/elicited (binary measure) (within subject) # of muscles with elicitable MEP	Data Type(s): Binary, Count Statistical Tests: Chi-Square, Fisher’s Exact Test
3. Electromyography (EMG) amplitude	Change in EMG amplitude in the presence of tSCS vs. no stimulation (within subject) Change in number of muscles with EMG output	Data Type(s): Continuous, Count Statistical Tests: Repeated Measures Analysis of Variance, Fisher’s Exact Test
Secondary outcomes (biomarker discovery)—Phase 1 and 2		
1. Neuronal (UCH-L1) and glial cell (GFAP) injury markers in blood	Changes in protein levels from baseline to post tSCS vs. sham	Data Type: Continuous Statistical Tests: Repeated Measures Analysis of Variance
2. CSF proteomics	Changes in protein levels from baseline to post tSCS vs. sham	Data Type: Continuous Statistical Tests: Non-linear Principal Components Analysis, followed by RM-ANOVA on PC scores
Secondary outcomes (patient-reported outcomes)—Phase 1 and 2		
3a. Incidence of neuropathic pain at 6-month follow-up 3b. Severity of neuropathic pain at 6-months follow-up	Modified DN4 questionnaire will be used to assess neuropathic pain at 6-months follow-up in tSCS vs. sham individuals (vs. historical TRACK-SCI participants)	Data Type: Integer, or Binarized at ≤ indicating neuropathic pain Statistical Test: Independent group Student’s *t*-test, or Chi-square
4. Quality of life	Neuro-QoL questionnaire will be used to assess patients’ quality of life at 6-months follow-up	Data Type: T score Statistical Test: Independent group Student’s *t*-test
5. Functional independence	Spinal Cord Independence Measure, Version 3 will be administered to assess functional independence at 6-months follow-up	Data Type: Integer (0–100) Statistical Test: Independent group Student’s *t*-test

### Harms

Potential harms associated with study participation include risks related to tSCS, biospecimen collection, and data privacy. Adverse events related to tSCS may include autonomic changes such as autonomic dysreflexia, manifested by blood pressure fluctuations, sweating, or headache, as well as skin irritation at electrode sites. Autonomic dysreflexia is less common in the acute phase of SCI but will be monitored closely. Although electrical stimulation in the acute phase of SCI could theoretically impair neurological recovery, this has not been observed in prior studies of chronic SCI. Risks associated with blood collection include transient pain, bruising, redness or swelling at the venipuncture site, infection, and rare episodes of fainting; blood will be drawn from existing intravenous lines whenever possible to minimize these risks. Cerebrospinal fluid sampling will be performed via an existing lumbar drain, accessing the drain carries a potential risk of infection, which will be mitigated through sterile technique. Risks related to privacy and confidentiality will be minimized through de-identification of data and biospecimens and secure storage procedures. Harms will be assessed systematically throughout the trial. Participants will be continuously monitored in the Neurointensive Care Unit, where neurological, cardiovascular, hematopoietic, abdominal, pulmonary, infectious, wound-related, and laboratory complications are routinely assessed as part of standard clinical care for acute spinal cord injury. Continuous telemetry and neurological examinations will be performed per standard of care. Vital signs will be recorded during tSCS sessions, along with surface electromyography recordings. The study team will document all vital signs and any vasoactive medications or infusions administered during stimulation sessions. All adverse events will be recorded by the study team and reviewed by the PI and the safety monitor. Events will be categorized by severity (mild, moderate, or severe) and assessed for their relationship to study participation (unrelated, possibly related, probably related, or definitely related). Serious adverse events and unanticipated adverse device effects will be reported in accordance with UCSF Institutional Review Board and sponsor requirements. Predefined stopping criteria include clinically significant hemodynamic instability, autonomic dysreflexia, deterioration in clinical status, and any serious adverse event judged to be related to the intervention.

### Participant timeline

Time schedule of enrollment, interventions, assessments, and visits for participants is shown in Figure 1.

### Sample size

Because no prior studies have evaluated tSCS during the acute phase of SCI to our knowledge, there are currently no directly comparable data available to support a conventional efficacy-based sample size calculation. Therefore, sample size estimates were informed by published studies in chronic SCI populations that employed similar stimulation paradigms and outcome measures. Power calculations on our preliminary patient data in children (*N* = 8) and adults (*N* = 15) (Sayenko et al., 2019) with chronic SCI suggest that the factorial within-subjects design yields sufficient power (1 − *β* > 0.80) to detect significant main effects of stimulation. Independent repeated-measures analysis of a recently published high-profile study (Moritz et al., 2024) that employed a similar transcutaneous stimulation paradigm (*N* = 60 participants) revealed that stimulation significantly improved ISNCSCI outcome measures over time (*p* < 0.05) with medium to large effect sizes and very high observed power (sensory score, eta squared effect size = 0.08, observed power = 1.0; motor score, eta squared effect size = 0.18, observed power = 0.97). To detect a difference with effect sizes as strong or stronger than seen in these analyses, with a power of 1 − *β* = 0.80 and alpha level set to 0.05. Although these effect sizes may not directly translate to the acute SCI setting, they provide a preliminary basis for study planning in the absence of acute SCI data. We will aim to enroll 15 subjects (*n* = 5 for Phase 1, and *n* = 10 for Phase 2). Given the expected heterogeneity of acute SCI and the small sample size, the study is not powered to detect modest between-group differences and efficacy analyses should be considered exploratory. Consistent with the early-phase safety and feasibility objectives of the study, efficacy outcomes will be used to estimate variability and effect sizes to inform future adequately powered clinical trials.

### Recruitment

We will prospectively identify potential subjects and enroll individuals presenting with ASIA A-C aSCI at ZSFG. Clinical research coordinators with formal training will begin screening patients upon admission to the emergency department and intensive care unit. We anticipate our sample size is feasible as ZSFG admits an average of 3–4 aSCI patients each month. Once a patient is confirmed to meet inclusion criteria, they will be approached for informed consent. Capacity to provide informed consent will be assessed prior to enrollment by experienced research study staff/physicians. Individuals with impaired decision-making capacity due to sedation, cognitive impairment, or acute medical illness will not be enrolled until the capacity is restored.

### Randomization

In Phase 2, subjects will be randomized to active or sham tSCS treatments using simple randomization, in a 1:1 ratio. The random allocation sequence will be generated by an independent study statistician using a computer-based randomization algorithm. Allocation concealment will be ensured using a centralized, computer-based randomization system accessible only at the time of assignment, thereby preventing foreknowledge of treatment allocation. Clinical research coordinators will enroll participants, and the treatment assignment will be implemented by a designated unblinded investigator responsible for administering the intervention. Baseline injury characteristics, including AIS grade, neurological level of injury, ISNCSCI scores, and MRI/BASIC score when available, will be summarized by treatment group and considered when interpreting preliminary efficacy outcomes.

### Blinding

Due to the objectives of the study, the identity of test and control treatments will be known to the investigator who administers treatment but will not be known to the research staff who conduct the assessments, the participants or the ICU care team/physicians. The following study procedures will be in place to ensure single-blind administration of study treatments: (1) access to the randomization code will be strictly controlled, (2) participants will be informed that they may or may not feel the stimulation, (3) the stimulator/its settings will be hidden from the assessor, (4) all the communication between the patient and investigator who will be monitoring for safety will be kept consistent between the sham and active treatment group. The study blind will be broken on completion of the clinical study and after the study database has been locked. Unblinding prior to study completion will only occur if required for clinical safety and will be conducted by the Principal Investigator with documentation of the reason and timing.

### Data collection methods

Once consent is obtained, coordinators will record all clinical variables and medical history in a secure, web-based portal (REDCap) in NINDS SCI Common Data Elements format. Within 72–96 h post-injury, patients will undergo baseline assessments including pain questionnaires [DN4 and International Spinal Cord Injury Pain Data Set (ISCIPDS)], International Standards for Neurological Classification of SCI (ISNCSCI) with electromyography (EMG), blood and CSF (if a lumbar drain is already in place for clinical purposes only) sampling for biomarkers. We anticipate that most, if not all, eligible subjects will have a lumbar drain as it is part of standard of care at our institution. Lower motor ISNCSCI will be repeated during the last 30 min of tSCS and at 7 days post-SCI (or at hospital discharge whichever is earlier). Surface EMG recordings obtained during active tSCS may contain stimulation-related artifacts. Appropriate signal processing procedures will be applied prior to analysis to reduce stimulation artifacts as necessary (Kim et al., 2021). Residual artifact contamination will be considered during interpretation of EMG findings.

For ISNCSCI and neurophysiological assessment, the participant will be instrumented with 10 surface electromyography recording electrodes (Delsys, Inc), placed bilaterally over the lower extremity muscles: Rectus Femoris (RF), Biceps Femoris (BF), Medial Gastrocnemius (MG), Tibialis Anterior (TA), Extensor Hallucis Longus (EHL). Stimulating electrodes will be placed on the skin over the low thoracic, lumbar spine and the grounding electrodes will be placed over the iliac crest bilaterally. The stimulation will be delivered as single, 1-ms monophasic square-wave pulses every 6 s while patient is lying in supine. Using 2-mA increments, the stimulation intensity will be increased from 2 to 100 mA or if the participant requests to stop stimulation. Three stimuli will be delivered at each intensity. The stimulation will be delivered using an experimental Neo-Stim 5 (Cosyma, LTD) transcutaneous spinal cord stimulator, as ARC-Ex currently does not support single-pulse tSCS paradigm required to evoked discrete motor responses. Neurophysiological outcome measures will include the presence or absence of elicitable motor evoked potentials (MEPs) and the number of muscles demonstrating elicitable responses (Table 1). The neurophysiological assessment will be performed at baseline twice (before and right after the first 60 min of continuous tSCS) and at 7 days post-SCI (or at hospital discharge whichever is earlier) if the standard of care continuous vital signs monitoring is still ongoing. Frequent vital signs will be captured during tSCS for analysis and will be monitored continuously as per standard of care.

When available, clinically acquired MRI data and intraoperative MEPs obtained as part of standard care will be reviewed. MRI severity will be characterized using the Brain and Spinal Injury Center (BASIC) score and used as an exploratory descriptor of injury severity and natural recovery potential (Talbott et al., 2015; Dhall et al., 2018).

We will measure glial fibrillary acidic protein (GFAP) and ubiquitin C-terminal hydrolase L1 (UCH-L1) in blood and CSF on the first day of stimulation prior to the first stimulation session (~72 h after injury). We will also measure these in blood on the final day of stimulation (treatment #5) or discharge, after last treatment, whichever is earlier. We will measure these in CSF on the final treatment before discharge, or before the lumbar drain is taken out if this happens before discharge, after treatment. GFAP and UCH-L1 will be assayed at ZSFG on the Abbott Alinity I Immunoassay system. At those same 2 time points we will also conduct a global discovery proteomic assay on both plasma and CSF. We will use the commercially available high-throughput protein biomarker discovery platform based on Olink’s Proximity Extension Assay (PEA), which reliably measures >5,400 proteins using <1 mL of biofluid.

At 6-month follow-up visit, participants will be asked about any adverse experiences and ISCNSCI with EMG assessment will be performed. We will also administer a modified DN4 and SCI Pain Basic Data Set questionnaire to assess presence of neuropathic or any other pain, the Spinal Cord Independence Measure (SCIM) to assess functional independence and Neuro-QoL (Lower Extremity function (Mobility) Short Form and Positive Affect and Well Being Short Form) to assess quality of life. See Figure 1 for a timeline of all study assessments.

### Retention

Participant retention will be supported through financial compensation for completion of inpatient and 6-month follow-up assessments, provision of transportation, and flexible scheduling including in-home visits when feasible. Participants who discontinue or deviate from the intervention will be encouraged to complete all outcome assessments. Outcome data collected will include neurological measures (ISNCSCI with electromyography), patient-reported outcomes (DN4, SCI Pain Data Set, Neuro-QoL, SCIM), and adverse events to allow continued evaluation of safety and clinical outcomes.

### Data management and monitoring

Clinical data will be collected on protocol-specific electronic Case Report Forms (eCRFs) using UCSF REDCap. Source data will be reviewed regularly by the site Principal Investigators for accuracy and completeness. Research staff will conduct routine checks to ensure timely entry, data integrity, and adherence to the protocol. Electromyography data will be recorded on UCSF/HIPAA compliant laptops and stored in the study specific UCSF Box folder.

Study conduct and participant safety will be monitored continuously by the study team, with immediate reporting of adverse events to the Principal Investigators and regular review of study progress, safety events, and protocol adherence. Adverse events will be recorded, graded, and reported to the UCSF Institutional Review Board and sponsor per regulatory requirements. No formal data monitoring committee is convened given the early-phase design and small sample size; oversight is provided by the investigators. A qualitative interim safety review will be conducted after Phase 1 prior to initiation of Phase 2.

### Analysis of primary and secondary efficacy outcome measures

For all participants, the primary outcome measure(s) will be: incidence of adverse events (number) for Phase 1 and 2 and change in ISNCSCI lower motor score over time for Phase 2. Secondary outcomes in both phases will be: ISNCSCI sensory scores, AIS grade, peak EMG amplitude, and mean EMG amplitude. For Phase I, change scores will be measured between baseline and post-trial, and in Phase II each measure will be taken across 5 trials. For all repeated measures analyses, we will use a factorial within-subjects repeated-measures analysis of variance (RM-ANOVA) to assess statistically significant change over time. This approach will allow us within a single statistical test to assess (1) the overall between-subjects effect of stimulation to determine the group differences between the stimulation and control groups, (2) the difference in changes over time between groups, and (3) the within-subjects effect of stimulation over trials, to determine the extent to which each individual changes over time. The RM-ANOVA procedure will be run for each outcome measure.

### Analysis of patient-reported secondary outcome measures

At 6-month follow-up, patient-reported outcome measures will be: neuropathic pain as measured by the DN4, quality of life as measured by the NeuroQoL, and functional independence measured by the SCIM. For DN4 data, incidence (count data) and severity (ordinal) will be assessed by Chi square and independent samples *T* test, respectively. NeuroQoL and SCIM measures will also be analyzed using independent samples *T* test.

### Analysis of biomarker discovery measures

Measures of UCHL-1 and GFAP protein will be taken over time and will be analyzed using repeated measures analysis of variance. To assess the big-data, high-dimensional proteomics from Aim 3, our statistical analysis plan consists of a two-tiered strategy of multivariate feature-extraction on multidimensional endpoints, followed by hypothesis testing for association with stimulation at the multivariate and univariate level. This approach will allow us to maximize power while minimizing N. Feature extraction on these metrics will be performed using non-parametric, non-linear principal component analysis (NL-PCA) followed by hypothesis testing with factorial within-subjects ANOVA, or non-parametric generalized estimating equation (GEE) as appropriate.

### Interim analysis

Prior to initiation of the Phase 2 component, a qualitative interim review of safety data from Phase 1 participants will be performed. This assessment will evaluate the type, frequency, and seriousness of adverse events to identify any emerging safety signals and to confirm that continuation to Phase 2 is appropriate. The results will be reviewed by the Principal Investigator and study team and reported to the IRB and in the quarterly progress reports to the Department of Defense.

## Confidentiality

Personal information of enrolled participants will be collected and maintained in secure, password-protected electronic systems compliant with institutional and HIPAA requirements. Access to identifiable data will be limited to authorized study personnel. Participants will be assigned unique study identification numbers, and identifiable information will be stored separately from research data. Data shared outside the study team, including for analysis or publication, will not contain direct identifiers. Regulatory authorities and the study sponsor may review identifiable records as required for monitoring or audit purposes. All records will be retained in accordance with institutional policy and federal regulations.

## Data sharing

De-identified individual participant data underlying the results reported in this study will be made publicly available through the Open Data Commons for Spinal Cord Injury (ODC-SCI; https://odc-sci.org) following publication of the primary results. Shared materials will include the final dataset, data dictionary, and relevant metadata necessary to reproduce the analyses. Data will be released under a Creative Commons Attribution 4.0 International (CC BY 4.0) license, permitting reuse, distribution, and adaptation with appropriate attribution. The code will be made available via GitHub.

## Dissemination policy

Upon study completion, aggregate study results will be made available through ClinicalTrials.gov, scientific conferences, and peer-reviewed publications. Participants who express interest in receiving study results will be provided with a plain-language summary of the overall study findings once data analysis is complete and results are publicly available. No individual participant results will be disclosed unless clinically indicated.
